# A novel synthetic microtubule inhibitor exerts antiproliferative effects in multidrug resistant cancer cells and cancer stem cells

**DOI:** 10.1038/s41598-021-90337-w

**Published:** 2021-05-24

**Authors:** Mina Park, Jee Won Hwang, Yena Cho, Saegun Kim, Sang Hoon Han, Jinsuh Yu, Sojung Ha, Woo-Young Kim, Su-Nam Kim, In Su Kim, Yong Kee Kim

**Affiliations:** 1 Research Institute of Pharmaceutical Sciences, College of Pharmacy, Sookmyung Women’s University, Seoul, 04310 Republic of Korea; 2 School of Pharmacy, Sungkyunkwan University, Suwon, 16419 Republic of Korea; 3 Natural Product Research Institute, Korea Institute of Science and Technology, Gangneung, 25451 Republic of Korea

**Keywords:** Cancer therapeutic resistance, Cancer stem cells, Microtubules, Mitotic spindle, Drug discovery

## Abstract

The success of cancer chemotherapy is limited by multidrug resistance (MDR), which is mainly caused by P-glycoprotein (P-gp) overexpression. In the present study*,* we describe a novel microtubule inhibitor, 5-(*N*-methylmaleimid-3-yl)-chromone (SPC-160002), that can be used to overcome MDR. A synthetic chromone derivative, SPC-160002, showed a broad spectrum of anti-proliferative effects on various human cancer cells without affecting P-gp expression and its drug efflux function. Treatment with SPC-160002 arrested the cell cycle at the M phase, as evidenced using fluorescence-activated cell sorting analysis, and increased the levels of mitotic marker proteins, including cyclin B, pS10-H3, and chromosomal passenger complex. This mitotic arrest by SPC-160002 was mediated by promoting and stabilizing microtubule polymerization, similar to the mechanism observed in case of taxane-based drugs. Furthermore, SPC-160002 suppressed the growth and sphere-forming activity of cancer stem cells. Our data herein strongly suggest that SPC-160002, a novel microtubule inhibitor, can be used to overcome MDR and can serve as an attractive candidate for anticancer drugs.

## Introduction

Microtubules, a principal component of the cytoskeleton, are composed of α-/β-tubulin heterodimers that play fundamental roles in mitosis, intracellular trafficking, and cellular structure^1,2^. During mitosis, the dynamic polymerization/depolymerization of spindle microtubules makes dividing cells sensitive to microtubule inhibitors (MTIs)^3^. MTIs are classified into two main groups: microtubule-destabilizing agents (such as vinblastine, colchicine, and combretastatin-A4) and microtubule-stabilizing agents (such as taxanes and epothilones)^4^. These microtubule-targeting agents have been broadly used against many solid and hematologic tumors; however, their clinical success is still largely determined by drug resistance^3,5^. In fact, an MTI, ixabepilone (a derivative of epothilone), which is capable of overcoming drug resistance, has been developed and approved by the FDA^6,7^.

Multidrug resistance (MDR), mainly caused by increased expression of cell-membrane ATP-binding cassette (ABC) transporters, is a crucial obstacle to cancer chemotherapy^8–10^. P-Glycoprotein (P-gp), also known as multidrug resistance protein 1 (MDR1 or ABCB1), acts as a major efflux pump for cytotoxic drugs, thus reducing intracellular drug concentrations^11,12^. Overexpression of P-gp is linked to MDR in many human cancer cells, including colon, kidney, adrenal, pancreas, and liver cancer cells^9,12,13^. P-gp has a broad spectrum of substrates, including topoisomerase inhibitors (i.e., etoposide and doxorubicin)^14^, microtubule-targeted drugs (i.e., vinblastine and paclitaxel)^15^, tyrosine kinase inhibitors (i.e., gefitinib and sunitinib), lipids, steroids, xenobiotics, peptides, and glucocorticoids^16^. Therefore, the development of a chemotherapeutic strategy to overcome MDR remains a major challenge in the treatment of cancer.

Chromones (4*H*-chomen-4-ones) and xanthones (9*H*-xanthen-9-ones) are oxygen-containing heterocyclic scaffolds that are widely distributed in nature and are known to have diverse biological activities, including anti-tumor, anti-microbial, anti-viral, anti-inflammatory, and anti-oxidant activities^17–19^. In particular, the bi- and tricyclic backbones in chromones and xanthones also exhibit biological activities, which differ depending on the type and/or location of the substituents^17–19^. Based on this variability of chromone and xanthone, a library of synthetic analogs of chromones and xanthones is being prepared to develop new bioactive agents^20^. In this study, we describe the molecular mechanism by which 5-(*N*-methylmaleimid-3-yl)-chromone (SPC-160002) inhibits the growth of various human cancer cells, including MDR cancer cells and cancer stem cells (CSCs). SPC-160002 suppressed the growth of not only a variety of human cancer cell lines but also P-gp-overexpressing MDR cancer cells, without affecting the expression and function of P-gp. These anti-proliferative effects were found to be mainly driven by mitotic arrest, resulting from stabilization of microtubule polymerization. In addition, SPC-160002 strongly suppressed the survival and sphere-forming activity of CSCs. Based on the established experimental results, we suggest the therapeutic potential of SPC-160002 as a novel microtubule-targeting drug that can overcome drug resistance.

## Results

### Maleimide-containing chromone derivatives show anti-proliferative effects in MDR cancer cells

**Figure 1:**
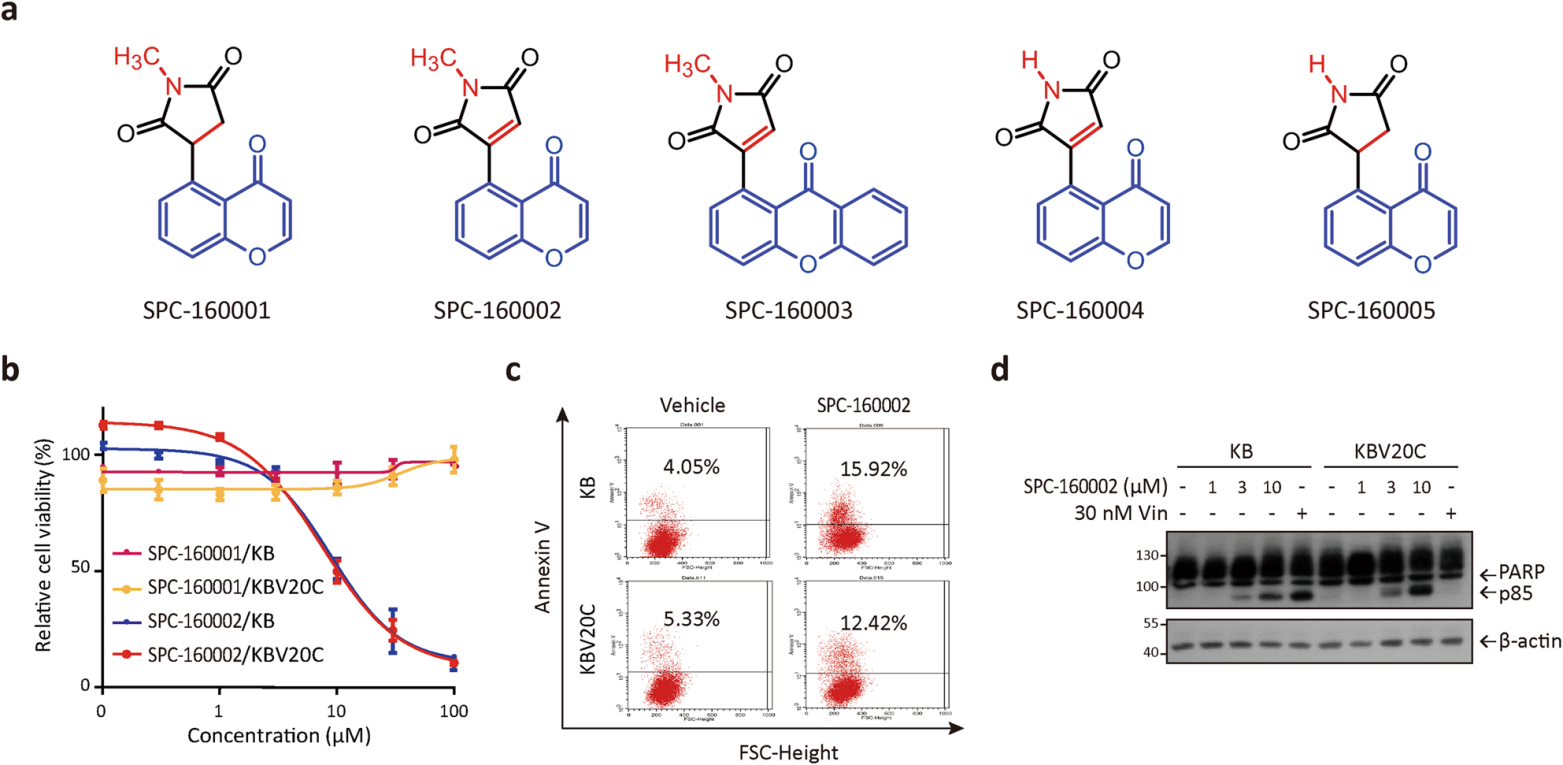
Maleimide-containing chromones inhibit proliferation of MDR cancer cells. (**a**) Structure of chromone or xanthone derivatives. SPC-160001: 1-methyl-3-(4-oxo-4*H*-chromen-5-yl)pyrrolidine-2,5-dione, SPC-160002: 1-methyl-3-(4-oxo-4*H*-chromen-5-yl)-1*H*-pyrrole-2,5-dione, SPC-160003: 1-methyl-3-(9-oxo-9*H*-xanthen-1-yl)-1*H*-pyrrole-2,5-dione, SPC-160004: 3-(4-oxo-4*H*-chromen-5-yl)-1*H*-pyrrole-2,5-dione, SPC-160005: 3-(4-oxo-4*H*-chromen-5-yl)pyrrolidine-2,5-dione. (**b**) The cytotoxicity of chromone and xanthone derivatives in KB and KBV20C cells was determined using MTT assay (48 h). Data is presented as mean ± SD (standard deviation, n = 3). (**c**) KB and KBV20C cells were treated with SPC-160002 (10 μM) for 24 h, followed by measurement of Annexin-V positive cells using flow cytometry. (**d**) Whole cell lysate from SPC-160002 or vincristine-treated cells (24 h) was subjected to immunoblotting.

To develop a novel anticancer drug that can overcome MDR, we examined the anti-proliferative effects of several chromone and xanthone derivatives (Fig. 1a) using P-gp-overexpressing KBV20C cells. The KBV20C cells, derived from KB^21,22^ were evaluated to have more than 300 times resistance to paclitaxel compared to its parental KB cells (Table 1). Among them, maleimide-containing chromones and xanthones (SPC-160002, SPC-160003, and SPC-160004) showed anti-proliferative effects in both KBV20C cells and their parental KB cells, while succinimide-containing compounds (SPC-160001 and SPC-160005) had no growth inhibition effect until a concentration of 100 μM in both the cells (Table 1 and Fig. 1b), indicating that the maleimide substituent is essential for the growth-inhibitory effect. Upon comparing the effects of SPC-160002, SPC-160003 and SPC-160004, we found that the structure of chromone had stronger activity than that of xanthone (Table 1). However, there was no difference in the anti-proliferative effects of *N*-methyl-substituted maleimide (SPC-160002) and (NH)-free-maleimide (SPC-160004) (Table 1). Altogether these results suggest that the chromones containing maleimide or 5-*N*-methylmaleimide exert anti-proliferative effects against MDR cancer cells. However, their potencies are much weaker than that of paclitaxel (Table 1).

**Table 1:** Cytotoxicity of maleimide-containing chomones and xanthone.

	IC_50_ (μM)	
	KB	KBV20C
SPC-160001	> 100	> 100
SPC-160002	10.3 ± 0.58	10.7 ± 1.03
SPC-160003	29.9 ± 4.12	33.5 ± 1.11
SPC-160004	6.59 ± 1.58	9.15 ± 1.67
SPC-160005	> 100	> 100
Paclitaxel (nM)	2.66 ± 1.29	> 1000

IC_50_ values of chromone and xanthone derivatives were calculated based on MTT assay at 48 h in KB and KBV20C cells. Data are mean ± SD (n = 3).

In this study, we also investigated the molecular mechanism by which SPC-160002 [5-(*N*-methylmaleimid-3-yl)-chromone] exerts anti-proliferative activity in MDR cancer cells. As shown in Table 2, SPC-160002 showed a broad spectrum of anti-proliferative effects on various human cancer cells. Based on the cytotoxicity of SPC-160002, we investigated whether SPC-160002 could induce apoptosis. Treatment of SPC-160002 led to an increase in the Annexin V-positive population in both KB and KBV20C cells (Fig. 1c). The apoptotic potential of SPC-160002 was further confirmed by measuring PARP cleavage, as a marker of apoptosis (Fig. 1d). As expected, KBV20C cells were found to be highly resistant to vincristine, thus showing the MDR phenotype of KBV20C cells (Fig. 1d). Taken together, our data show that SPC-160002 has a broad spectrum of anti-proliferative effects on various human cancer cells as well as MDR cancer cells.

**Table 2:** Anti-proliferative effects of SPC-160002 on various human cancer cells.

	Cell lines	IC_50_ (μM)
Lung cancer	H226B	5.82 ± 1.93
	A549	14.29 ± 3.25
Prostate cancer	LNCaP	3.74 ± 2.04
	DU145	13.33 ± 1.22
Breast cancer	SK-BR-3	3.52 ± 1.34
	MCF7	26.89 ± 0.86
Colorectal cancer	DLD-1	11.52 ± 1.58
	HCT116	11.6 ± 1.68
Glioblastoma	A172	7.67 ± 2.08
	U87MG	10.93 ± 1.50
Liver cancer	HepG2	7.04 ± 1.72

IC_50_ values of SPC-160002 were calculated for each cell line using MTT assay. Data are mean ± SD (n = 3).

### SPC-160002 does not affect P-gp expression and its drug efflux function

**Figure 2:**
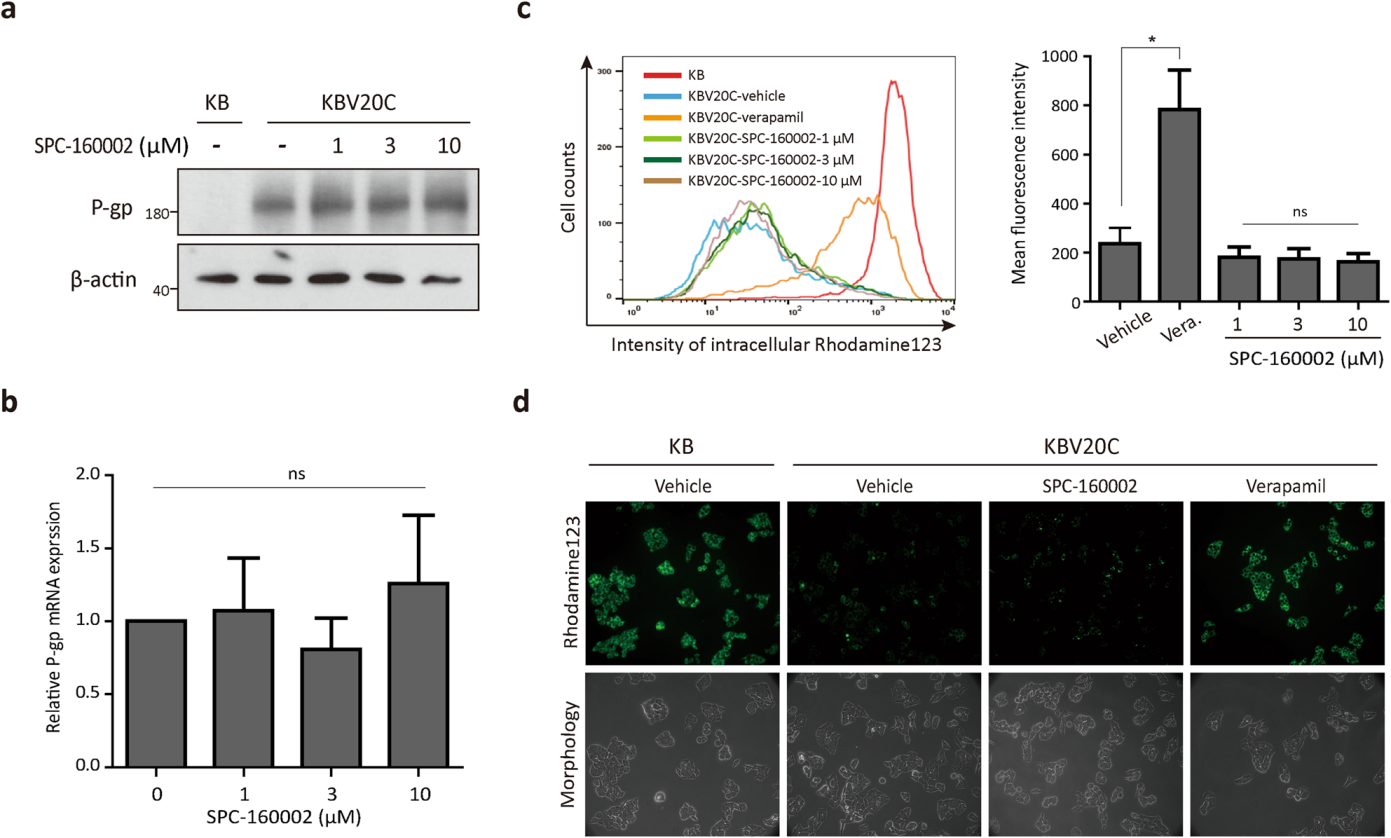
SPC-160002 does not affect P-gp expression and its drug efflux function. (**a**) The P-gp protein levels in KB and SPC-160002-treated KBV20C cells (24 h) were analyzed. (**b**) KBV20C cells were treated with SPC-160002 for 24 h, followed by determination of P-gp mRNA using quantitative RT PCR. Data is presented as mean ± SD (n = 3). (**c**, **d**) KB and KBV20C cells were treated with DMSO (vehicle), 10 μM verapamil (vera) or SPC-160002 for 1 h followed by 10 μM rhodamine 123 for 3 h. Intracellular rhodamine 123 was measured using (**c**) flow cytometry or (**d**) fluorescence microscopy. Representative image of SPC-160002-treated group (10 μM) is shown. Error bars represent SD (n = 3). *p < 0.05.

To determine whether SPC-160002 affects the expression or function of P-gp, we first examined the expression levels of P-gp mRNA and protein. As shown in Fig. 2a,b, there were no differences in both mRNA and protein levels between the control and SPC-160002-treated cells. Following that, to investigate whether the efflux function of P-gp is affected by SPC-160002, we measured the intracellular accumulation of rhodamine 123, a well-established substrate of P-gp. Rhodamine 123 was found to be extensively accumulated in KB cells, but not in KBV20C cells (Fig. 2c), re-validating the MDR phenotype of KBV20C cells. There was no change in the accumulation of rhodamine 123 in KBV20C cells upon SPC-160002 treatment, while there was a profound increase upon treatment with a P-gp inhibitor, verapamil (Fig. 2c), which was further confirmed using fluorescence microscopy (Fig. 2d). These results indicate that SPC-160002 does not affect both P-gp expression and its drug efflux function.

### SPC-160002 blocks cell cycle progression at the G_2_/M phase

**Figure 3:**
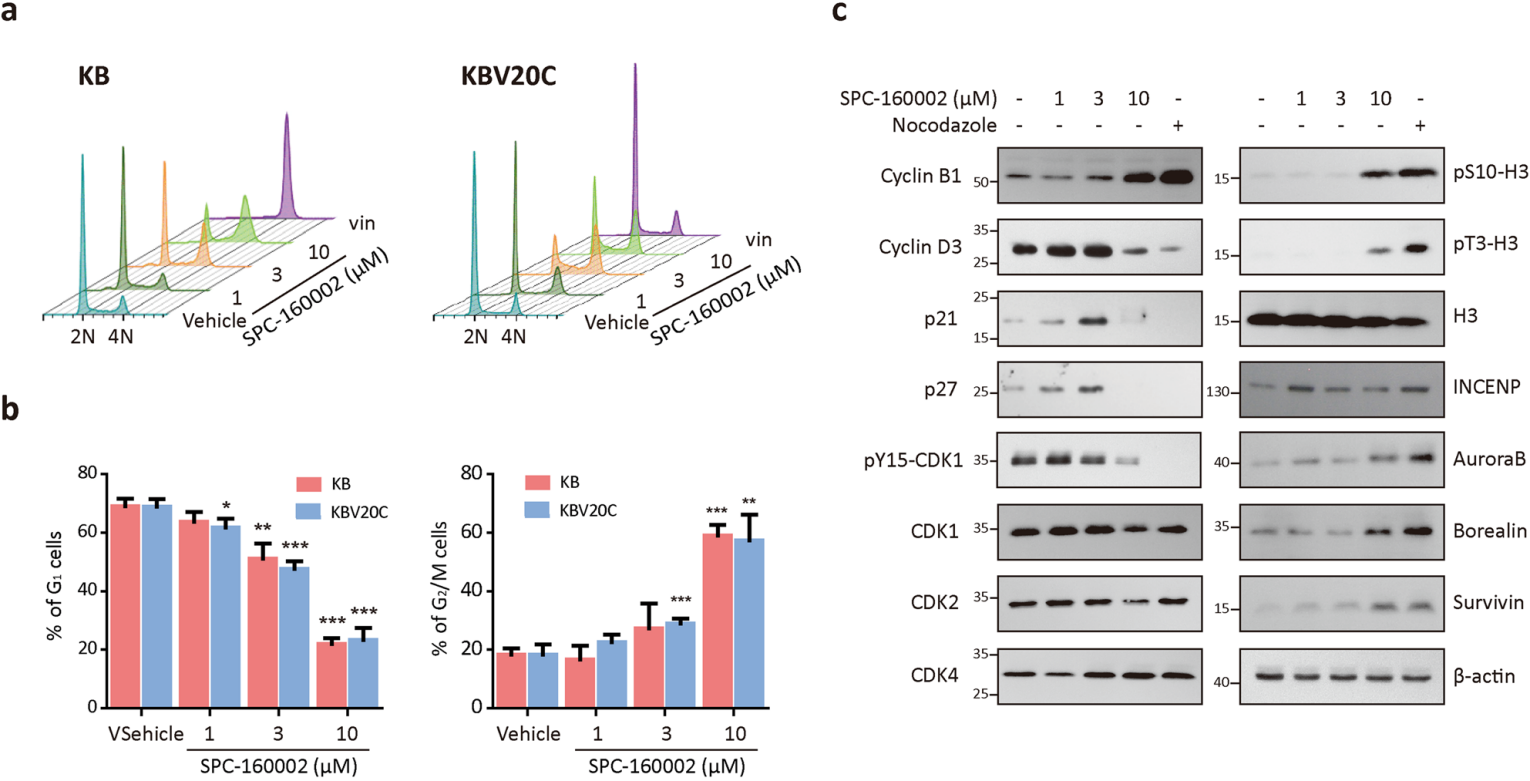
SPC-160002 arrests cell cycle progression at the G_2_/M phase. (**a**) KB and KBV20C cells were treated with DMSO (vehicle), SPC-160002 or 30 nM vincristine (vin) for 24 h and then stained with PI. DNA contents were analyzed using flow cytometry. (**b**) Percentage of cells in the G_1_ or G_2_/M phases in (**a**) has been expressed as a bar graph. Data from 3 independent experiments were presented as mean ± SD. *p < 0.05, **p < 0.01 and ***p < 0.001. (**c**) KB cells were treated with SPC-160002 or 100 ng mL^-1^ nocodazole for 24 h and then the whole cell lysates were subjected to immunoblotting.

To investigate the molecular mechanisms by which SPC-160002 exerts anti-proliferative activity on cancer cells, we analyzed the cell cycle using flow cytometry. As shown in Fig. 3a,b, treatment with SPC-160002 dose-dependently decreased the proportion of cells in G_1_ phase and increased the proportion of cells in G_2_/M phase in both KB and KBV20C cells. As expected, vincristine induced G_2_/M arrest in KB cells, but not in KBV20C cells. To confirm G_2_/M arrest by SPC-160002, we examined the levels of cell cycle-related proteins. SPC-160002 dramatically increased cyclin B1, a key regulator of the G_2_/M transition, but decreased G_1_/S-specific cyclin D3 (Fig. 3c). In addition, there was a drastic decrease in the level of pY15-cdk1, an inactive form, indicating that SPC-160002 promotes M phase transition from G_2_ phase. There was a clear decrease in the levels of the cdk inhibitors, p21 and p27 at 10 µM of SPC-160002, similar to the effect seen in case of nocodazole, a well-known mitotic inhibitor. Furthermore, SPC-160002 treatment dramatically increased both pS10-H3 and pT3-H3, which are representative histone marks for M phase (Fig. 3c). It has been well-established that pS10-H3 is mainly generated by Aurora B kinase in chromosomal passenger complex (CPC) during mitosis^23^, and the CPC complex, composed of Aurora B, INCENP, Borealin, and Survivin, is a major regulator of chromosome segregation during mitosis and cytokinesis^24–26^. We thus examined the expression levels of CPC components in the presence of SPC-160002. As expected, SPC-160002 treatment dramatically increased the level of all CPC components, Aurora B, Borealin, and Survivin, with only the exception of INCENP. These expression profiles upon SPC-160002 treatment were consistent with those of nocodazole. Altogether, our data support that SPC-160002 is a mitotic inhibitor and causes mitotic arrest of cancer cells.

### SPC-160002 blocks mitotic exit in cell cycle

**Figure 4:**
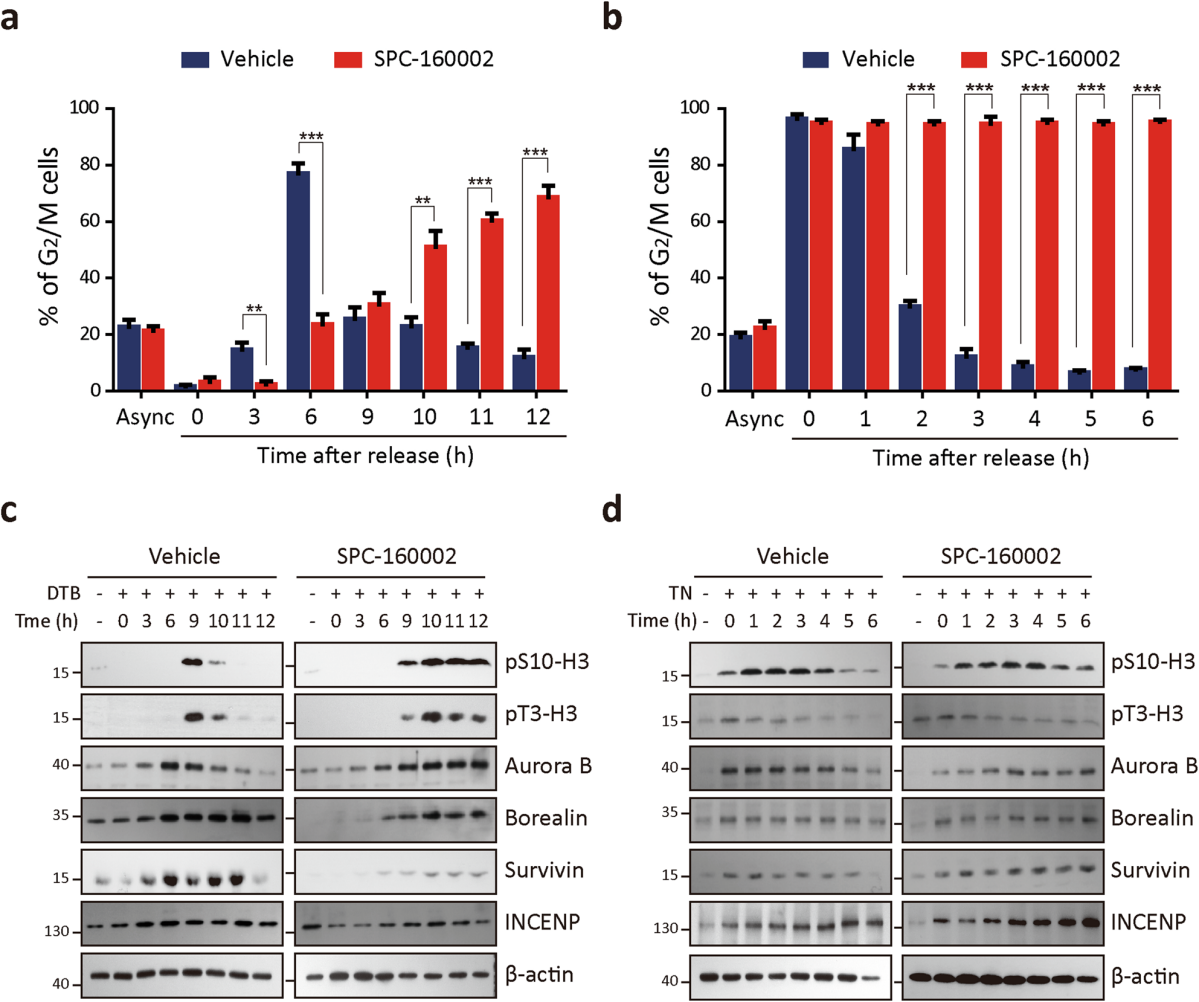
SPC-160002 blocks mitotic exit. (**a**–**d**) KB cells were synchronized using DTB (**a**, **c**) or TN (**b**, **d**) block and then released with or without 10 μM SPC-160002 for the indicated times. (**a**, **b**) Cells were stained with PI and analyzed using flow cytometry. Percentage of cells in G_2_/M phase has been shown. Error bars indicate SD of three independent replicates. **p < 0.01 and ***p < 0.001. (**c**, **d**) Whole cell lysates corresponding to (**a**, **b**) were examined using immunoblotting.

To explore the molecular mechanism underlying M phase arrest by SPC-160002, we analyzed the kinetics of cell cycle progression. KB cells were synchronized at the G_1_-S boundary using double thymidine block (DTB) and then released in the absence or presence of SPC-160002. Six hours after release, most of the control cells moved to the G_2_/M phase and then went back to the G_1_ phase after 9 h of release, while only 20% of SPC-160002-treated cells were in the G_2_/M phase after 6 h of release; the cells in the G_2_/M phase very slowly increased and accumulated after 12 h of release (Supplementary Figs. 1a and 4a). These results indicate that SPC-160002 retarded the cell cycle progression from G_1_ to M phase and blocked the exit from M phase, leading to mitotic arrest. To further evaluate the blockade of M phase exit by SPC-160002, we synchronized cells at pro-metaphase using thymidine-nocodazole (TN) block and then released them with or without SPC-160002 treatment. In the control group, most of the cells moved to the G_1_ phase within 2 h, whereas SPC-160002-treated cells remained at the M phase at 6 h (Supplementary Figs. 1b and 4b), further supporting the blockade of mitotic exit by SPC-160002. Next, we examined the changes in the levels of cell cycle markers using cell lysates corresponding to flow cytometry experiments. There was a transient increase in the levels of pS10-H3, pT3-H3, and CPC in the control cells at the M phase, followed by a rapid decrease at the G1 phase, which depends on cell cycle progression (Fig. 4c). However, the levels in SPC-160002-treated cells were sustained after 12 h of release (Fig. 4c). Furthermore, similar results were obtained from the TN-release experiment; the levels of pS10-H3, pT3-H3, and CPC in the SPC-160002 treated cells were higher than those in the control cells (Fig. 4d). These results were in good accordance with the previous results of cell cycle progression (Supplementary Fig. 1a,b and 4a,b), strongly supporting that SPC-160002 blocks mitotic exit in the cell cycle, and subsequently, causes mitotic arrest.

### SPC-160002 promotes and stabilizes tubulin polymerization

**Figure 5:**
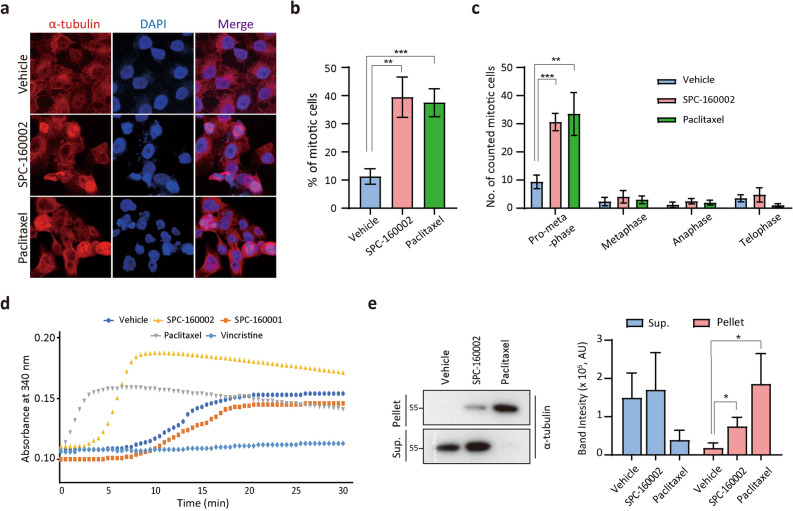
SPC-160002 promotes and stabilizes microtubule polymerization. (**a**–**c**) KB cells were treated with DMSO (vehicle), 10 μM SPC-160002 or 10 nM paclitaxel for 24 h and then immunostained with α-tubulin. Representative images are shown in (**a**). (**b**) Cells displaying mitotic morphology were counted. Error bars represent SD (n = 3). (**c**) The cells counted in (**b**) were categorized into pro-metaphase, metaphase, anaphase, and telophase. Data from 3 independent experiments were presented as bar chart (mean ± SD). (**d**) In vitro tubulin polymerization assay with DMSO (vehicle), 10 μM SPC-160002, 10 μM SPC-160001, 100 nM paclitaxel, or 30 nM vincristine. (**e**) KB cells were treated with DMSO (vehicle), 10 μM SPC-160002 or 100 nM paclitaxel for 24 h. Supernatant (sup) and pellet of cell lysate were examined using immunoblotting with a β-tubulin antibody. Data obtained from three replicated experiments were quantified and graphed. *p < 0.05, **p < 0.01, and ***p < 0.001.

Given the mitotic arrest by SPC-160002, we performed immunofluorescence staining of microtubules to analyze the effect of SPC-160002 on mitotic morphology. Remarkably, 39% of SPC-160002-treated cells had a mitotic spindle structure with strong microtubule fluorescence intensity, which was comparable to paclitaxel treated cells (Fig. 5a,b). We then subdivided the cells displaying mitotic morphology according to the mitotic substages (Supplementary Fig. 2). As shown in Fig. 5c, in the presence of SPC-160002, there was a profound increase in the mitotic cell populations in pro-metaphase, but not in anaphase or telophase, similar to the phenotypes of taxane class drugs^27,28^. The association of taxanes with β-tubulin stabilizes the microtubule structure and consequently suppresses the microtubule dynamics, resulting in mitotic arrest^27,28^. To examine the effects of SPC-160002 on tubulin polymerization dynamics, we performed an in vitro microtubule polymerization assay. Purified tubulin was incubated with SPC-160002 and the absorbance at 340 nm was measured every 30 s during incubation. Remarkably, we found that SPC-160002 dramatically promoted tubulin polymerization (Fig. 5d), like paclitaxel. Indeed, there was polymerization and stabilization of intracellular microtubule upon SPC-160002 treatment (Fig. 5e). However, the microtubule stabilizing effect of SPC-16002 appears to be much weaker than that of paclitaxel. Altogether, these results strongly suggest that SPC-160002 is a novel microtubule inhibitor that promotes and stabilizes microtubule polymerization.

### SPC-160002 inhibits survival and sphere-formation of cancer stem cells

It has been well established that CSCs represent a small population of cells with tumor-initiation potential^29,30^. Because CSCs are resistant to conventional chemotherapeutic agents as well as radiation therapy, and play a role in tumor metastasis and relapse^29,30^, they serve as important targets for the development of novel anticancer drugs. The resistance of CSCs to chemotherapy is mainly due to the overexpression of ABC transporters^8,31^.

**Figure 6:**
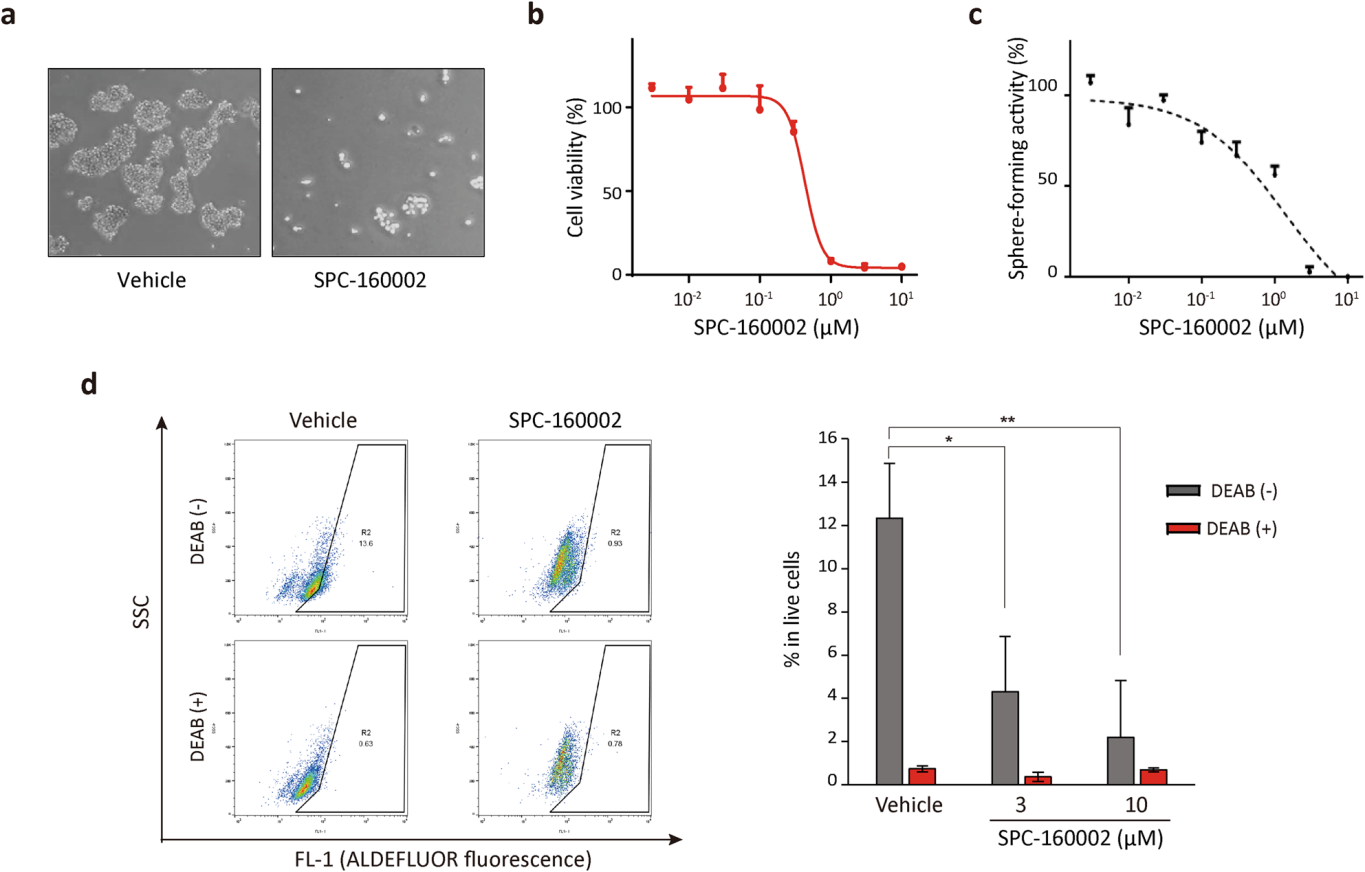
SPC-160002 inhibits survival and sphere-formation ability of CSCs. (**a**) The KB cells established CSC spheres in CSC enriched culture. (**b**) The cytotoxicity of KB cells cultured in the attached condition with or without SPC-160002 (4 days) supplemented with 1% FBS was determined by MTS assay. Error bars represent SD (n = 6). (**b**) CSC cultured KB cells were re-seeded in poly-HEMA-coated 96-well plates for 4 days with or without SPC-160002 with CSC medium, followed by counting of the number of spheres (≥ 100 μm in diameter). Data represent mean ± SD (n = 4). (**c**) The secondary sphere cells cultured with or without SPC-160002 for 48 h were dissociated into single cells and then incubated with Aldefluor substrate, with or without DEAB. The Aldefluor-positive cells were detected using flow cytometry. Error bars indicate SD of 3 independent replicates. *p < 0.05 and **p < 0.01.

We cultured KB cells in the attachment free condition with CSC culture medium. It generated typical CSC spheres (Fig. 6a) and were maintained in multiple passages. The P-gp expression of CSC spheres is higher than that of 2D monolayer cultured cells (Supplementary Fig. 3), which is in good accordance with the observation that the KB-derived CSCs are at least tenfold more resistant to paclitaxel than the bulk culture KB cells (Supplementary Fig. 4). In the presence of SPC-160002, both the proliferation of 2D monolayer cultured KB cells and CSC sphere-forming activity were dramatically inhibited with the similar potency (Fig. 6b,c). To further confirm the inhibitory effect of SPC-160002 on the survival of CSCs, specifically, we performed Aldefluor assay which quantify aldehyde dehydrogenase (ALDH, a CSC-predominant enzyme)-positive CSCs. In the presence of SPC-160002, there was a significant reduction in the ALDH-positive cells in the KB-derived CSC spheres (Fig. 6d), suggesting that SPC-160002 effectively eliminates the CSCs in KB cells. To further strengthen the anti-CSC-sphere forming activity of SPC-160002, we used U87 glioblastoma CSC model in which the role of P-gp in CSC was well characterized^32^. As expected, SPC-160002 treatment effectively inhibits the population of CSCs in U87 cells (Supplementary Fig. 5).

## Discussion

Several MTIs, including paclitaxel and vincristine, have been used as potent and effective chemotherapeutics for various cancer treatments; however, acquired resistance to these molecules due to MDR serves as a major impediment for successful treatment. Thus, development of microtubule-targeting drugs that can overcome MDR is a major research problem. It has been reported that several chromone compounds have the ability to target microtubules^33,34^; however, there is no evidence whether these anti-microtubule chromones have MDR-overcoming ability. Herein, we describe that SPC-160002 [5-(*N*-methylmaleimid-3-yl)-chromone] is a novel microtubule inhibitor that exerts anti-proliferative effects in both MDR cancer cells and CSCs. SPC-160002 is effective in human cancer cells derived from various tissues; moreover, it effectively inhibits cell growth in MDR cancer cells. It is assumed that SPC-160002 is not a substrate for ABC transporters including P-gp, because it strongly suppresses the proliferation of P-gp-expressing cancer cells, resulting in apoptotic cell death. In addition, it does not affect the expression and drug efflux function of P-gp. Although SPC-160002 has limited selectivity and superiority over MDR cancer cells, it can serve as an attractive candidate for the development of potent anticancer drugs with the ability to overcome MDR. The presence of CSCs in tumor makes them resistant to chemotherapy and radiation therapy, consequently leading to recurrence and metastasis of cancer^29,30^. Although extensive research has been devoted to the development of anticancer drugs that target CSCs^29,31^, the effective treatment of CSCs remains a challenge that needs to be addressed. SPC-160002 inhibits the sphere-forming activity of both KB and U87 glioblastoma cells and decreases the ALDH-positive cells in CSC-like spheres, suggesting the potential of SPC-160002 as a CSC-targeting drug.

Chromones and xanthones are classes of heterocyclic compounds that commonly exist in nature^17–19^. They consist of diverse functional substituents that are implicated in a variety of pharmacological activities, including anti-inflammatory, anti-bacterial, anti-oxidant, and anti-tumor activities. Their numerous biological and pharmacological activities depend on the structural composition of the substituents and several of these derivatives are indeed used as therapeutic agents. Our data show that SPC-160002 stabilizes microtubule structure and induces mitotic arrest, subsequently leading to apoptosis. There are the obvious differences between SPC-160002 and paclitaxel on microtubule stabilization effect (Fig. 5c,d), which seems to be due to their potency differences. The potency of paclitaxel is over 300 times higher than that of SPC-160002 (Table 1), which is good agreement with the observations that microtubule stabilizing effect of paclitaxel is higher than that of SPC-16002 (Fig. 5c,d). The microtubule-stabilizing effect of SPC-160002 appears to be due to the maleimide group, because the succinimide-containing compounds (SPC-160001 and SPC-160005) did not show any such effect, while similar effects were seen in maleimide-containing compounds (SPC-160003 and SPC-160004). Although we cannot currently explain how the maleimide group contributes to microtubule stabilization, it is possible that the maleimide group interacts with microtubules by the Michael-type addition reaction. Tubulin is a sulfhydryl-rich heterodimer with 20 cysteine residues, which reacts with sulfhydryl-directed reagents, thus regulating microtubule polymerization or depolymerization^35,36^. This explanation is further supported by our preliminary observation that SPC-160002 blocks irreversibly cell proliferation (Supplementary Fig. 6). Since our SPC compounds are less potent or effective than paclitaxel, their pharmacological properties are needed to be improved for further developing these compounds as anti-cancer drugs. Further research, including the number, position, or variation of the maleimide substituent, is needed to improve their potencies or efficacies. Altogether, we suggest that SPC-160002 might be an attractive lead molecule for further development of chemotherapeutics with the ability to overcome MDR.

## Materials and methods

### Typical procedure for the reaction of chromone (SPC-160001 and SPC-160005)

1-Methyl-1*H*-pyrrole-2,5-dione (44.4 mg, 0.4 mmol, 200 mol%) and DCE (1 mL) were added to an oven-dried sealed tube charged with 4*H*-chromen-4-one (29.2 mg, 0.2 mmol, 100 mol%), [RhCp*Cl_2_]_2_ (3.1 mg, 0.005 mmol, 2.5 mol%), AgSbF_6_ (6.8 mg, 0.02 mmol, 10 mol %), and PivOH (20.4 mg, 0.2 mmol, 100 mol %) under air at room temperature. The reaction mixture was stirred for 20 h at 80 °C, then diluted with EtOAc (3 mL), and finally concentrated in vacuo. The residue was purified using flash column chromatography (EtOAc/*n*-hexanes = 2:1) to obtain 48.9 mg of SPC-160001 at a yield of 95%, the structural analysis data for which is shown in the “Supplementary Information”.

### Typical procedure for the reaction of chromone and xanthone (SPC-160002, SPC-160003, and SPC-160004)

1-Methyl-1*H*-pyrrole-2,5-dione (44.4 mg, 0.4 mmol, 200 mol %) and DCE (2 mL) were added to an oven-dried sealed tube charged with 4*H*-chromen-4-one (29.2 mg, 0.2 mmol, 100 mol %), [Ru(*p*-cymene)Cl_2_]_2_ (12.2 mg, 0.02 mmol, 10 mol %), AgNTf_2_ (31.0 mg, 0.08 mmol, 40 mol %), and AgOAc (60.1 mg, 0.6 mmol, 300 mol %) under air at room temperature. The reaction mixture was stirred for 12 h at 120 °C, then diluted with EtOAc (5 mL), and finally concentrated in vacuo. The residue was purified using flash column chromatography (EtOAc/*n*-hexanes = 3:1) to obtain 43.7 mg of SPC-160002 at a yield of 86%, the structural analysis data for which is shown in the “Supplementary Information”.

### Cell culture

Human cancer cell lines used in this study (KB, H226B, A549, LNCaP, DU145, SK-BR-3, MCF7, DLD-1, HCT116, A172, and HepG2) were purchased from American Type Culture Collection (Manassas, VA, USA). The multidrug-resistant cell line KBV20C was derived from KB^21,22^. KB, KBV20C, H226B, A549, LNCaP, DU145, and DLD-1 cells were grown in RPMI-1640 (HyClone Laboratories, Logan, UT, USA), while SK-BR-3, MCF7, HCT116, A172, and HepG2 cells were grown in DMEM (HyClone Laboratories), both supplemented with 10% FBS (HyClone Laboratories) and 100 units/mL of penicillin/streptomycin (HyClone Laboratories). KBV20C cells were maintained with 20 nM vincristine in their cell growth medium. Glioblastoma multiform, U87 cells, a kind gift from Dr. Hyunggee Kim (Korea University), were cultured in DMEM/F12 medium (Invitrogen, Waltham, MA). All cells were maintained at 37 °C under 5% CO_2_ in a humidified cell culture incubator.

### Chemicals and antibodies

Verapamil, vincristine, and paclitaxel were purchased from Sigma-Aldrich (St. Louis, MO, USA). The commercial antibodies used included anti-β-actin (sc-47778, dilution of 1:10,000), anti-CDK1 (sc-54, dilution of 1:1000), anti-cyclin B1 (sc-752, dilution of 1:1000), and α-tubulin (sc-23948, dilution of 1:5000) antibodies purchased from Santa Cruz Biotechnology (Dallas, TX, USA); anti-phospho-H3-S10 (#9701, dilution of 1:1000), anti-phospho-CDK1-Y15 (#4539, dilution of 1:1000), anti-CDK2 (#2546, dilution of 1:1000), anti-CDK4 (#12790, dilution of 1:1000), and anti-H3 (#9715, dilution of 1:5000) antibodies purchased from Cell Signaling Technology (Danvers, MA, USA); anti-INCENP (ab12183, dilution of 1:1000), anti-survivin (ab76424, dilution of 1:1000), anti-aurora B (ab2254, dilution of 1:1000), and anti-phospho-H3-T3 (ab78351, dilution of 1:1000) antibodies purchased from Abcam (Cambridge, UK); and anti-borealin (NBP1-89951, dilution of 1:1000) antibody purchased from Novus Biologicals (Centennial, CO, USA). HRP-conjugated secondary antibodies (dilution of 1:10,000) were purchased from Jackson ImmunoResearch Laboratories (West Grove, PA).

### Cell synchronization

To arrest cells in the G_1_/S phase, KB and KBV20C cells at 30% confluence were maintained in growth media supplemented with 2 mM thymidine for 18 h. After washing twice with PBS, the cells were switched to fresh growth media for 9 h. Next, 2 mM thymidine was re-added to the culture and cells were treated with it for 15 h. To arrest cells in the mitotic phase, cells at 40% confluence were incubated with 2 mM thymidine for 24 h. After 24 h, cells were washed twice with PBS and incubated with fresh media for 3 h, followed by addition of and incubation with 100 ng mL^-l^ nocodazole for 12 h.

### Western blot analysis

Cells were lysed using NP-40 lysis buffer (20 mM Tris–HCl pH 8.0, 137 mM NaCl, 10% glycerol, 1% non-diet P-40, 2 mM EDTA) supplemented with 1× protease and phosphatase inhibitor cocktails (Roche, Basel, Switzerland). The protein concentration of the lysate was then quantified using Bradford assay (Bio-Rad, Hercules, CA, USA), according to the manufacturer’s instructions. Equal amounts of proteins were subjected to SDS-PAGE and transferred to a PVDF membrane (Millipore, Billerica, MA, USA). The membranes were blocked with 5% (w/v) skim milk in TBS containing 0.1% Tween 20 (TBS-T) for 1 h and then incubated with the primary antibody overnight at 4 °C. After washing three times with TBS-T, the membrane was incubated with a secondary antibody for 1 h at room temperature. The bound antibodies were visualized using chemiluminescent detection reagents (Advansta, Menlo Park, CA, USA). The intensity of all protein bands was quantified using image processing software (Image Studio version 5.0, LI-COR Biotechnology, Lincoln, NE).

### Quantitative reverse transcription PCR

The cellular RNA was extracted using TRIsure (Bioline, London, UK) and 1 μg of total RNA was reverse-transcribed to cDNA using the SensiFAST cDNA synthesis kit (Bioline). P-gp mRNA level was analyzed via quantitative PCR using SensiFAST SYBR No-ROX Kit (Bioline) and Eco Real-Time PCR System (Illumina, San Diego, CA). Data were normalized to transcripts encoding GAPDH or L32. The sequences of primer set for P-gp were 5′-CTTCAGGGTTTCACATTTGGC-3′ and 5′-GGTAGTCAATGCTCCAGTGG-3′; for GAPDH were 5′-CTCATGACCACAGTCCATGCCATC-3′ and 5′-CTGCTTCACCACCTTCTTGATGTC-3′; for L32 were 5′-CAACATTGGTTATGGAAGCAACA-3′ and 5′-TGACGTTGTGGACCAGGAACT-3′. Reaction parameters were as follows: cDNA synthesis at 37 °C for 60 min, transcriptase inactivation at 85 °C for 5 min, and PCR cycling at 95 °C for 10 s, 58 °C for 20 s, and 72 °C for 20 s (40 cycles).

### Flow cytometry analysis

Synchronized KB and KBV20C cells were washed with cold PBS and then fixed using pre-chilled 70% ethanol for at least 1 h on ice. After washing with PBS, the fixed cells were treated with 0.2 mg mL^−1^ RNase at 37 °C for 1 h, followed by addition of 10 μg mL^−1^ propidium iodide (BD Biosciences, Franklin Lakes, NJ, USA), and finally analyzed on a FACS Calibur system (BD Biosciences) using the FlowJo software.

### Immunofluorescence

Cells seeded on coverslips were fixed by incubation with pre-chilled 99% methanol at − 20 °C for 30 min. After fixation, cover slips were blocked with PBS-BT (1% BSA, 0.1% Triton X-100 in PBS) for 30 min at room temperature. Subsequently, coverslips were incubated with anti-tubulin antibody diluted in PBS-BT overnight at 4 °C and then with Alexa Fluor 594-conjugated secondary antibodies for 1 h at room temperature. After 4,6-diamidino-2-phenylindole (DAPI; Thermo Fisher Scientific, Waltham, MA, USA) staining for 3–5 min, the cells were washed three times with PBS and mounted onto glass slides. Staining was evaluated using a Zeiss 710 immunofluorescence microscope (Carl Zeiss, Oberkochen, Germany) and ZEN software.

### Rhodamine 123 uptake assay

Rhodamine 123 uptake assay was performed as previously described^22^. KB and KBV20C cells were pretreated with verapamil or SPC-160002 for 1 h and then incubated with 10 μM rhodamine 123 (Sigma-Aldrich) for 3 h under normal culture conditions. After washing with cold PBS, cells were resuspended in PBS just before analysis. Fluorescence intensity of intracellular rhodamine 123 was determined using flow cytometry.

### In vitro microtubule polymerization assay

Tubulin Polymerization Kits (Cytoskeleton, Inc., Denver, CO, USA) were used according to the manufacturer’s instructions. Briefly, 5 μL of vehicle (dimethyl sulfoxide; DMSO) or compound was mixed with porcine tubulin (3 mg mL^-1^) in a reaction buffer (80 mM 1,4-piperazinediethanesulfonic acid (PIPES), 0.5 mM ethylene glycol-bis(β-aminoethyl ether)-*N*,*N*,*N′*,*N′*-tetraacetic acid (EGTA), 1 mM guanosine triphosphate (GTP), 2 mM MgCl_2_, 10% glycerol, made up to a final volume of 100 μL) in wells of a 96-well plate. While incubating at 37 °C, the absorbance at 340 nm was measured every 60 s for 1 h using a microplate reader (SpectraMax i3x, Molecular Devices, San Jose, CA, USA).

### Separation of tubulin into polymerized and unpolymerized fractions

Whole cell lysates were taken from 60 mm culture dishes with 250 μL of lysis buffer (10 mM Tris–HCl pH 7.4, 100 mM NaCl, 1 mM EDTA, 1 mM EGTA, 1% NP-40, 10% glycerol) at 4 °C for 20 min. A centrifuge was used at 13,000 rpm for 10 min to separate soluble and insoluble fractions of cell lysates. The soluble and insoluble fraction were collected, and the pellet was resuspended in 15 μL of lysis buffer and 5 μL of 5X sample buffer (60 mM Tris–HCl pH 6.8, 25% glycerol, 2% SDS, 5% 2-mercaptoethanol, 0.1% bromophenol blue). Supernatant and pellet fractions were then loaded for SDS-PAGE, followed with analysis by western blotting.

### CSC sphere culture

The surfaces of culture plates were covered using poly-HEMA (poly-2-hydroxyethyl methacrylate, Sigma-Aldrich) dissolved at a concentration of 5 mg mL^-1^ in 95% ethanol. After drying completely, the cells were seeded and incubated with neurosphere-culture media [DMEM/F12 medium (Invitrogen) containing 20 ng mL^-1^ epidermal growth factor, basic fibroblast growth factor, and B27 neural supplement (Invitrogen), in addition to 100 units mL^-1^ penicillin/streptomycin]. After 4 days, the enriched primary CSC spheres were dissociated to single cells with 0.5 mg mL^-1^ Dispase (Thermo Scientific) to generate secondary spheres for measuring drug effect.

### CSC sphere-formation inhibition assay

To assess the effect on CCS sphere-forming ability, the cells (1000 cells per well) derived from the primary CSC cultures were re-seeded into poly-HEMA-coated 96-well plate and treated with or without drug. After 4 days, the spheres were fixed in 1% formalin and the spheres (≥ 100 μm in diameter) were counted under microscope (Nikon, Japan). For comparison, the cytotoxicity to the cells cultured in the attached condition on the plates in same duration were determined using MTS assay. Culture medium supplemented with 10% or 1% FBS was used to minimize the effect of serum proteins in the action of drugs because the defined CSC medium contains minimal proteins.

ALDH assay was performed to quantify putative CSC cells that express high ALDH. After the secondary spheres were cultured with or without SPC-160002 for 48 h, the cells were dissociated and analyzed with Aldeflour assay kit (Stem Cell Technology, Cambridge, MA) followed by flow cytometry. A well-defined ALDH inhibitor, *N*,*N*-diethylaminobenzaldehyde (DEAB), was used for negative control.

### Statistical analysis

All statistical analysis was performed by using GraphPad Prism 8.0. The data are presented as mean ± SD (standard deviation) from at least three independent experiments as indicated in each figure legend. Comparison between data from two groups was analyzed by using a two tailed unpaired Student’s *t*-test for independent samples and *p* value < 0.05 were considered statistically significant. *p < 0.05, **p < 0.01, and ***p < 0.001.

## Supplementary Information

Supplementary Information.
