# Supplementary material for: A novel synthetic microtubule inhibitor exerts antiproliferative effects in multidrug resistant cancer cells and cancer stem cells
Source: Sci Rep. 2021 May 24;11:10822. doi: 10.1038/s41598-021-90337-w (PMC8144389; doi:10.1038/s41598-021-90337-w)

Supplementary Information

A novel synthetic microtubule inhibitor exerts antiproliferative effects in multidrug resistant cancer cells and cancer stem cells

Mina Park^1,#^, Jee Won Hwang^1,#^, Yena Cho^1^, Saegun Kim^2^, Sang Hoon Han^2^, Jinsuh Yu^1^, Sojung Ha^1^, Woo-Young Kim^1^, Su-Nam Kim^3^, In Su Kim^2,^ *, and Yong Kee Kim^1,^*

^1^Research Institute of Pharmaceutical Sciences, College of Pharmacy, Sookmyung Women’s University, Seoul 04310, Republic of Korea

^2^School of Pharmacy, Sungkyunkwan University, Suwon 16419, Republic of Korea

^3^Natural Product Research Institute, Korea Institute of Science and Technology, Gangneung 25451, Republic of Korea

**Synthesis and characterization of compounds**

The compounds used in this study were synthesized using typical chemical procedures described in the ‘Methods’ section. Characterization data of these compounds, including ^1^H NMR and ^13^C NMR spectra, are given as follows:

**1-Methyl-3-(4-oxo-4*H*-chromen-5-yl)pyrrolidine-2,5-dione (SPC-160001)**

48.9 mg (95%); White solid; mp=205.6-208.3°C; ^1^H NMR (400 MHz, DMSO-d_6_) δ 8.27 (d, *J*=5.8 Hz, 1H), 7.76 (t, *J*=7.5 Hz, 1H), 7.66 (d, *J*=8.1 Hz, 1H), 7.39 (d, *J*=6.7 Hz, 1H), 6.30 (d, *J*=5.9 Hz, 1H), 4.42 (br s, 1H), 3.04-2.98 (m, 1H), 2.90 (s, 3H), 2.54 (dd, *J*=17.3, 6.3 Hz, 1H); ^13^C{^1^H} NMR (100 MHz, DMSO-d_6_) δ 177.7, 177.4, 176.5, 157.7, 156.0, 136.4, 133.7, 130.8, 121.6, 119.1, 113.0, 47.0, 36.9, 24.4; IR (KBr) υ 3080, 2924, 2853, 1774, 1692, 1641, 1603, 1478, 1434, 1340, 1279, 1117, 1029, 770, 687 cm^-1^; HRMS (orbitrap, ESI) calcd for C_14_H_12_NO_4_ [M+H]^+^ 258.0766, found 258.0761.

**1-Methyl-3-(4-oxo-4*H*-chromen-5-yl)-1*H*-pyrrole-2,5-dione (SPC-160002)**

43.7 mg (86%); White solid; mp=198.6-200.3°C; ^1^H NMR (400 MHz, DMSO-d_6_) δ 8.33 (d, *J*=6.0 Hz, 1H), 7.88 (t, *J*=8.4 Hz, 1H), 7.81 (d, *J*=9.6 Hz, 1H), 7.39 (d, *J*=8.0 Hz, 1H), 6.84 (s, 1H), 6.33 (d, *J*=6.0 Hz, 1H), 2.94 (s, 3H); ^13^C NMR (100 MHz, DMSO-d_6_) δ 176.3, 170.7, 169.2, 156.7, 156.3, 149.0, 133.8, 128.7, 127.3, 124.6, 122.5, 120.6, 112.8, 23.7; IR (KBr) υ 3081, 2900, 2734, 1745, 1698, 1632, 1598, 1471, 1421, 1358, 1296, 1103, 1036, 780 cm^-1^; HRMS (quadrupole, EI) calcd for C_14_H_9_NO_4_ [M]^+^ 255.0532, found 255.0533.

**1-Methyl-3-(9-oxo-9*H*-xanthen-1-yl)-1*H*-pyrrole-2,5-dione (SPC-160003)**

58.8 mg (96%); White solid; mp=233.9-236.0°C; ^1^H NMR (400 MHz, CDCl_3_) δ 8.22 (d, *J*=9.6 Hz, 1H), 7.78.6 Hz, 1H), hit7.65 (t, *J*=9.2 Hz, 1H), 7.38 (t, *J*=8.0 Hz, 1H), 7.21 (d, *J*=8.4 Hz, 1H), 6.51 (s, 1H), 3.15 (s, 3H); ^13^C NMR (100 MHz, CDCl_3_) δ 176.9, 170.8, 169.5, 156.7, 155.6, 150.1, 135.2, 134.2, 130.2, 126.8, 125.7, 124.5, 124.3, 122.0, 120.5, 120.4, 117.7, 24.2; IR (KBr) υ 2982, 2937, 1771, 1692, 1649, 1601, 1475, 1401, 1348, 1222, 1126, 927 cm^-1^; HRMS (quadrupole, EI) calcd for C_18_H_11_NO_4_ [M]^+^ 305.0688, found 305.0685.

**3-(4-Oxo-4*H*-chromen-5-yl)-1*H*-pyrrole-2,5-dione (SPC-160004)**

15.8 mg (32%); Yellow solid; mp=263.9–265.2°C; ^1^H NMR (400 MHz, DMSO-d_6_) δ 10.94 (s, 1H), 8.32 (d, *J*=6.0 Hz, 1H), 7.87 (t, *J*=8.4 Hz, 1H), 7.80 (d, *J*=8.4 Hz, 1H), 7.38 (d, *J*=7.2 Hz, 1H), 6.70 (s, 1H), 6.33 (d, *J*=6.0 Hz, 1H); ^13^C NMR (100 MHz, DMSO-d_6_) δ 176.3, 172.0, 170.5, 156.6, 156.3, 149.6, 133.7, 128.8, 127.3, 125.4, 122.5, 120.4, 112.9; IR (KBr) υ 3062, 2911, 2821, 1768, 1713, 1655, 1325, 1211, 1178, 786 cm^-1^; HRMS (quadrupole, EI) calcd for C_13_H_7_NO_4_ [M]^+^ 241.0375, found 241.0375.

**3-(4-Oxo-4*H*-chromen-5-yl)pyrrolidine-2,5-dione (SPC-160005)**

31.6 mg (65%); Light yellow solid; mp=261.2-263.1°C; ^1^H NMR (500 MHz, DMSO-d_6_) δ 11.05 (br s, 1H), 8.26 (d, *J*=5.9 Hz, 1H), 7.74 (t, *J*=7.5 Hz, 1H), 7.63 (d, *J*=8.4 Hz, 1H), 7.35 (d, *J*=7.2 Hz, 1H), 6.29 (d, *J*=5.9 Hz, 1H), 4.42 (br s, 1H), 2.92 (br s, 1H), 2.56 (dd, *J*=17.3, 6.7 Hz, 1H); ^13^C NMR (125 MHz, DMSO-d_6_) δ 178.6, 177.8, 177.7, 157.6, 155.9, 136.7, 133.7, 130.7, 121.7, 118.9, 113.1, 48.5, 38.2; IR (KBr) υ 3060, 2924, 2856, 1777, 1710, 1648, 1340, 1270, 1178, 741 cm^-1^; HRMS (orbitrap, ESI) calcd for C_13_H_10_NO_4_ [M+H]^+^ 244.0609, found 244.0605.

**^1^H NMR and ^13^C NMR spectra of all products (SPC-160001 ~ SPC-160005)**

- **Table S1. Antibodies for Western and immunostaining analysis**

| **Antibody** | **Species** | **Isotype** | **Company** | **Cat. No.** | **Dilution** |
| --- | --- | --- | --- | --- | --- |
| β-actin | Mouse | IgG_1_ | Santa Cruz Biotechnology | sc-47778 | 1:10,000 |
| CDK1 | Mouse | IgG_2a_ | Santa Cruz Biotechnology | sc-54 | 1:1,000 |
| cyclin B1 | Rabbit | IgG | Santa Cruz Biotechnology | sc-752 | 1:1,000 |
| α-tubulin | Mouse | IgG_1_ | Santa Cruz Biotechnology | sc-23948 | 1:5,000 |
| p-H3-S10 | rabbit | - | Cell Signaling Technology | #9701 | 1:1,000 |
| p-CDK1-Y15 | Rabbit | - | Cell Signaling Technology | #4539 | 1:1,000 |
| CDK2 | Rabbit | - | Cell Signaling Technology | #2546 | 1:1,000 |
| CDK4 | Rabbit | IgG | Cell Signaling Technology | #12790 | 1:1,000 |
| H3 | Rabbit | - | Cell Signaling Technology | #9715 | 1:5,000 |
| INCENP | Rabbit | IgG | Abcam | ab12183 | 1:1,000 |
| survivin | Rabbit | IgG | Abcam | ab76424 | 1:1,000 |
| aurora B | Rabbit | IgG | Abcam | ab2254 | 1:1,000 |
| p-H3-T3 | Rabbit | - | Abcam | ab78351 | 1:1,000 |
| borealin | Rabbit | IgG | Novus Biologicals | - NBP1-89951 | 1:1,000 |
| Anti-mouse-HRP | Goat | - | Jackson ImmunoResearch | 111-035-003 | 1:10,000 |
| Anti-rabbit-HRP | Goat | - | Jackson ImmunoResearch | 115-035-003 | 1:10,000 |


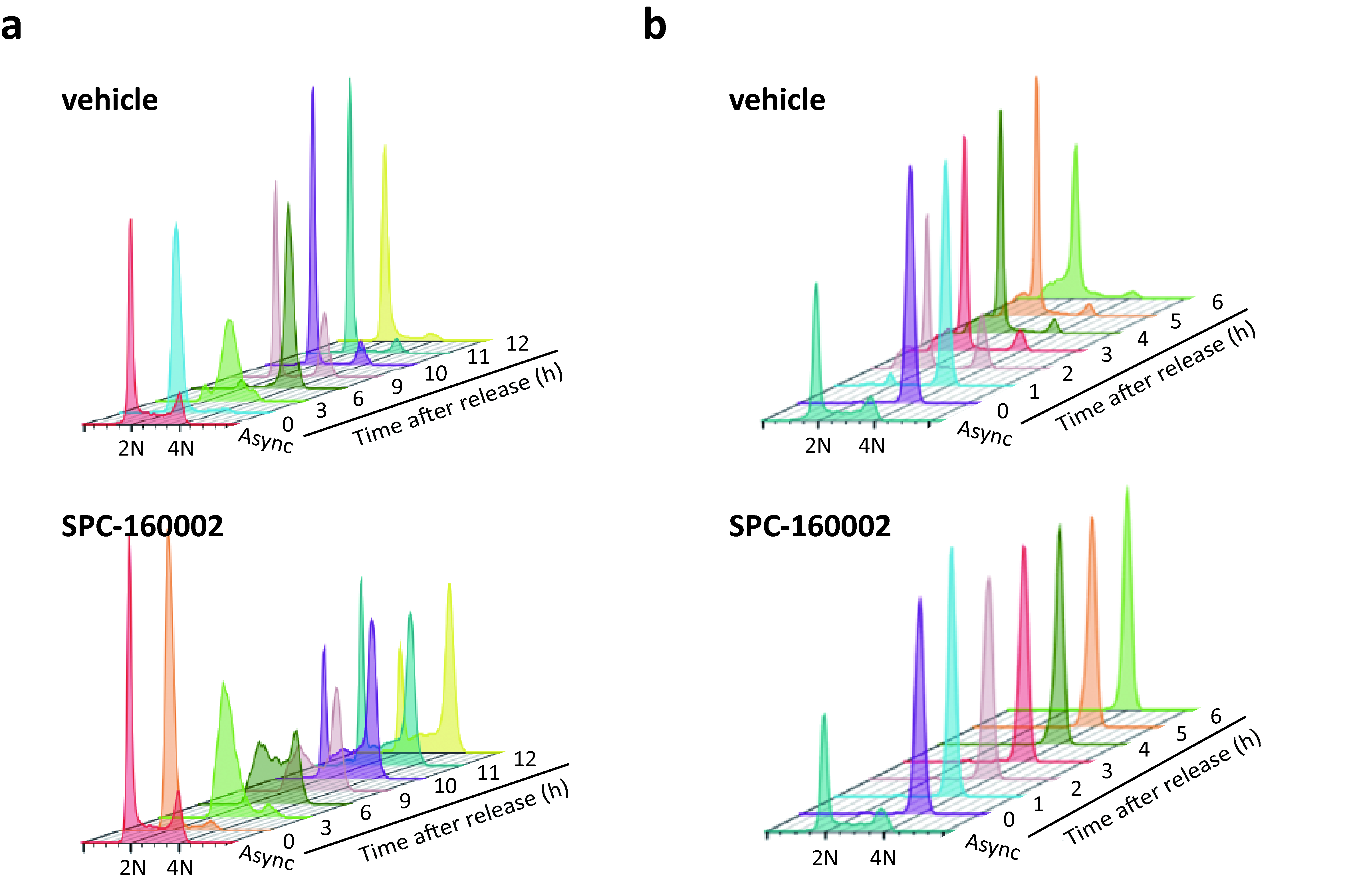


**Supplementary Figure 1. SPC-160002 blocks mitotic exit.**

KB cells were synchronized using DTB (**a**) or TN block (**b**) and then released with or without SPC-160002 for the indicated times. Cells were stained with PI and analyzed using flow cytometry. DTB, double thymidine block; TN, thymidine nocodazole; PI, propidium iodide


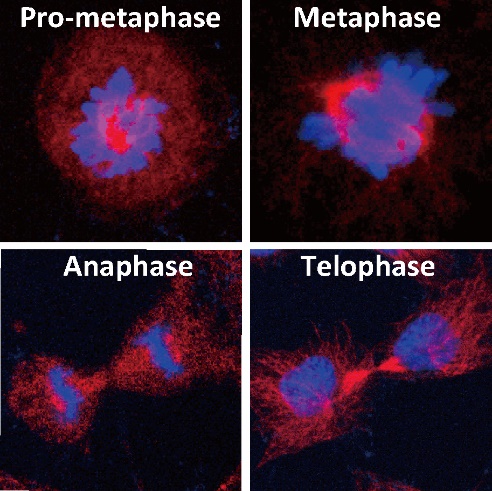


**Supplementary Figure 2. Morphology of mitotic phase.**

Microtubule and chromosome morphology that classified the phases of mitosis in Figure 5c.


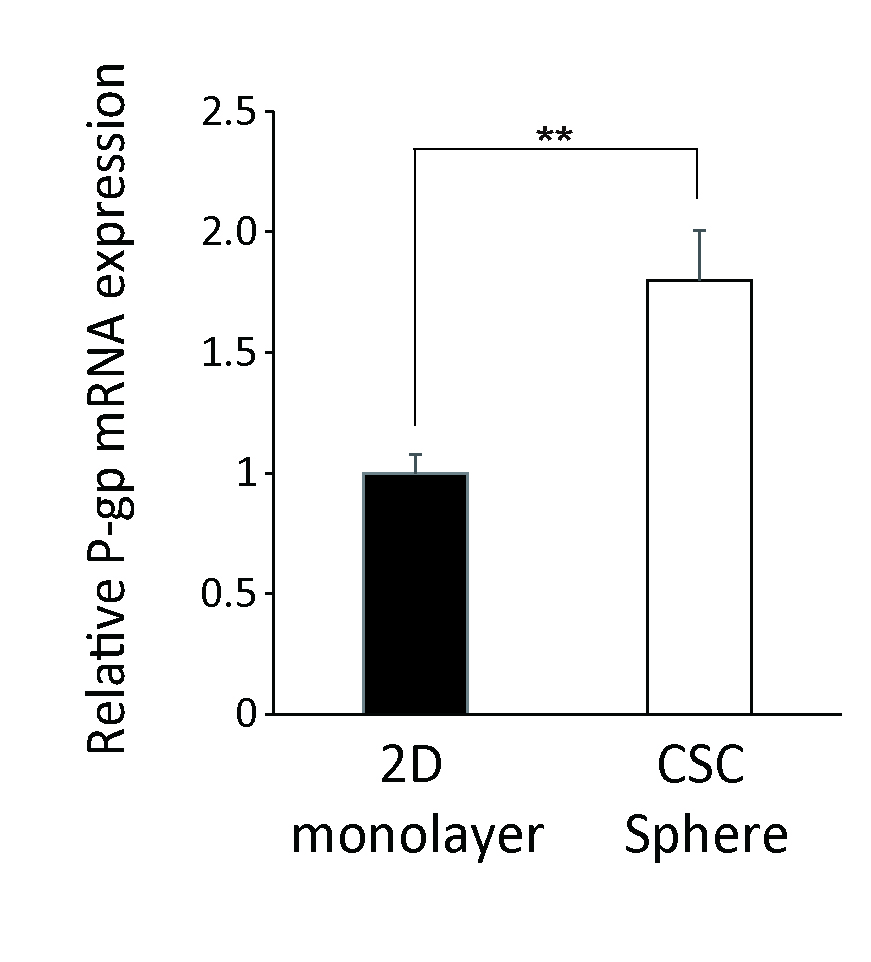


**Supplementary Figure 3. P-gp expression increases in KB CSC spheres than in 2D monolayer culture.**

Quantitative RT PCR presented P-gp mRNA is highly detected in the KB CSC sphere cells than in the 2D monolayer cultured cells. Data represent mean ± SD (n=4, **: *p* < 0.01).

**
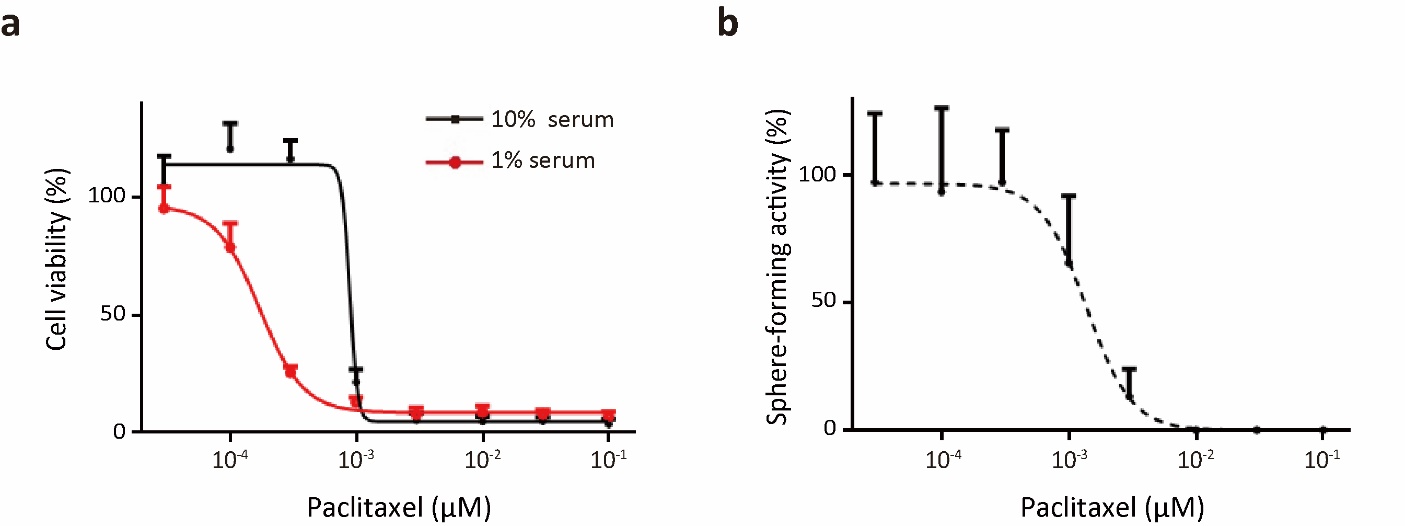
**

**Supplementary Figure 4. The KB-derived CSCs are resistant to the P-gp substrate paclitaxel.**

(**a**) The cytotoxicity of KB cells cultured in the attached condition with or without paclitaxel (4 days) supplemented with FBS (1% or 10%) was determined by MTS assay. Error bars represent SD (n=6). (**b**) CSC cultured KB cells were re-seeded in poly-HEMA-coated 96-well plates for 4 days with or without paclitaxel with CSC medium, followed by counting of the number of spheres (≥ 100 μm in diameter). Data represent mean ± SD (n=4).

Since the effect of paclitaxel can be diminished by serum albumin and the CSC culture medium contains minimal protein, the 1% FBS condition may be relevant to compare with CSC culture The KB-derived CSCs have shown to be at least 10-fold more resistant to paclitaxel the bulk culture cells, indicating the MDR phenotype of the CSCs.


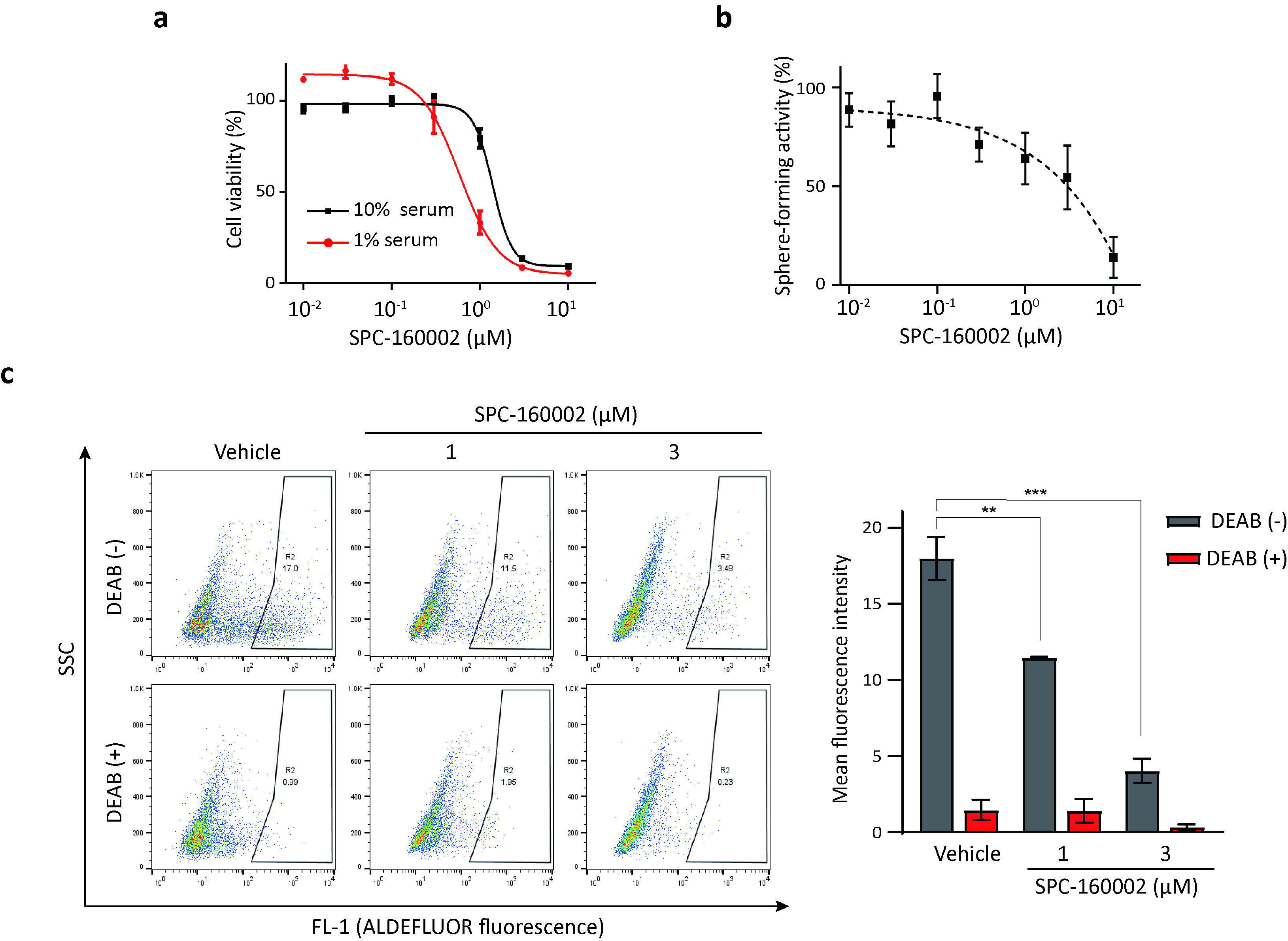


**Supplementary Figure 5. SPC-160002 inhibits survival and sphere-formation ability of CSCs of a glioblastoma multiform cell line U87.**

(**a**) The cytotoxicity of U87 cells cultured in the attached condition with or without the drug (4 days) was determined by MTT assay. Error bars represent SD (n=6). (**b**) CSC cultured U87 cells were re-seeded in poly-HEMA-coated 96-well plates for 4 days with or without the drug, followed by counting of the number of spheres. Data represent mean ± SD (n=6). (**c**) The secondary sphere cells were dissociated into single cells and then incubated with Aldefluor substrate, with or without DEAB. The Aldefluor-positive cells were detected using flow cytometry. Error bars indicate SD of 3 independent replicates. **: *p* < 0.01 and ***: *p* < 0.001.

**
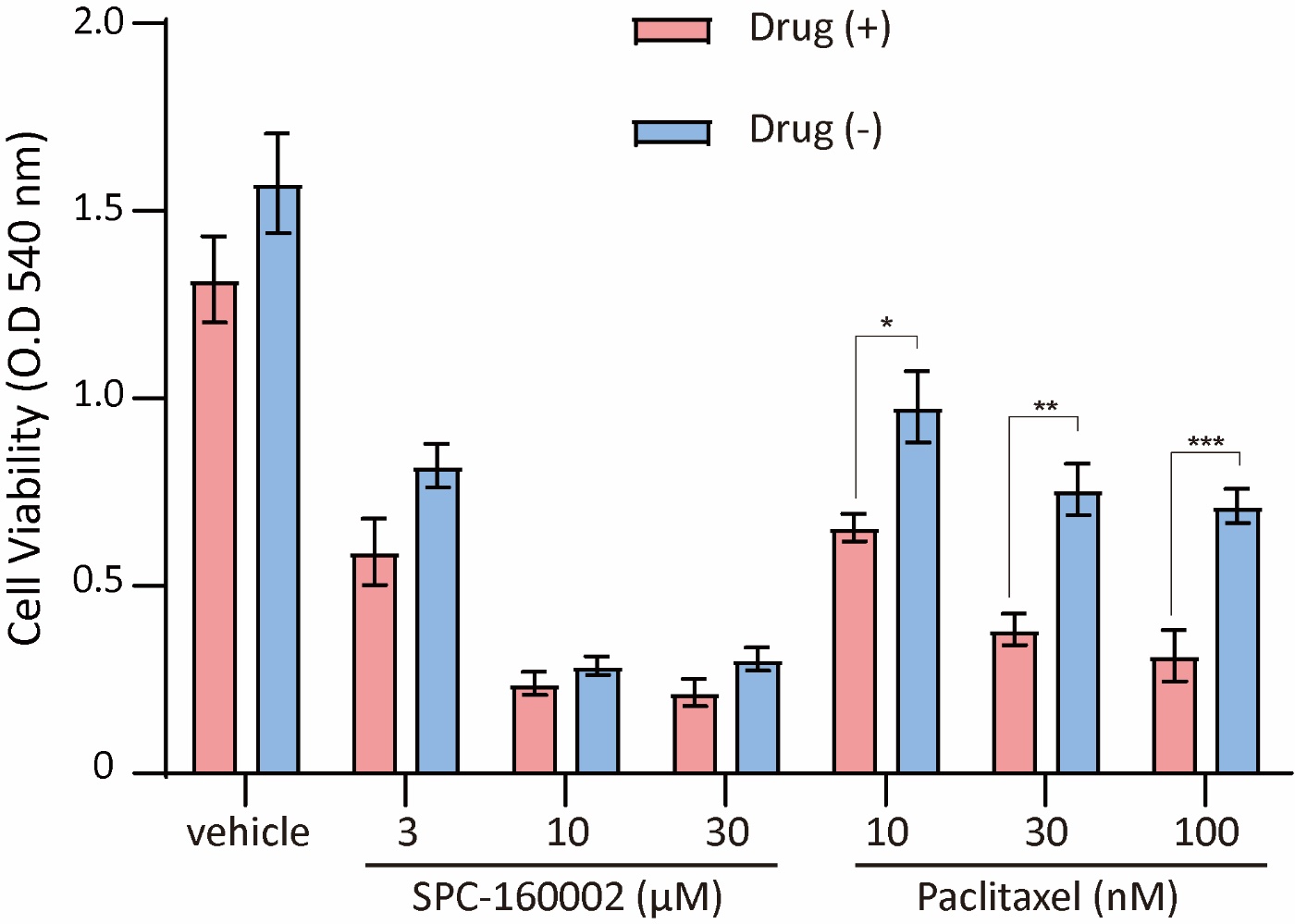
**

**Supplementary Figure 6. SPC-60002 blocks irreversibly the cell proliferation.**

After 24 h treatment of KB cells with each drugs (SPC-160002 or paclitaxel), the cells were incubated for additional 48 h with or without the drugs. And cell viabilities were measured by MTT assay. The cell proliferation was completely blocked even when SPC-160002 was removed from the media, but in the case of paclitaxel, the cells began to grow again when it was removed. These results strongly indicate that SPC-60002 blocks irreversibly the cell proliferation. Error bars indicate SD of 3 independent replicates. *: *p* < 0.05, **: *p* < 0.01, and ***: *p* < 0.001.


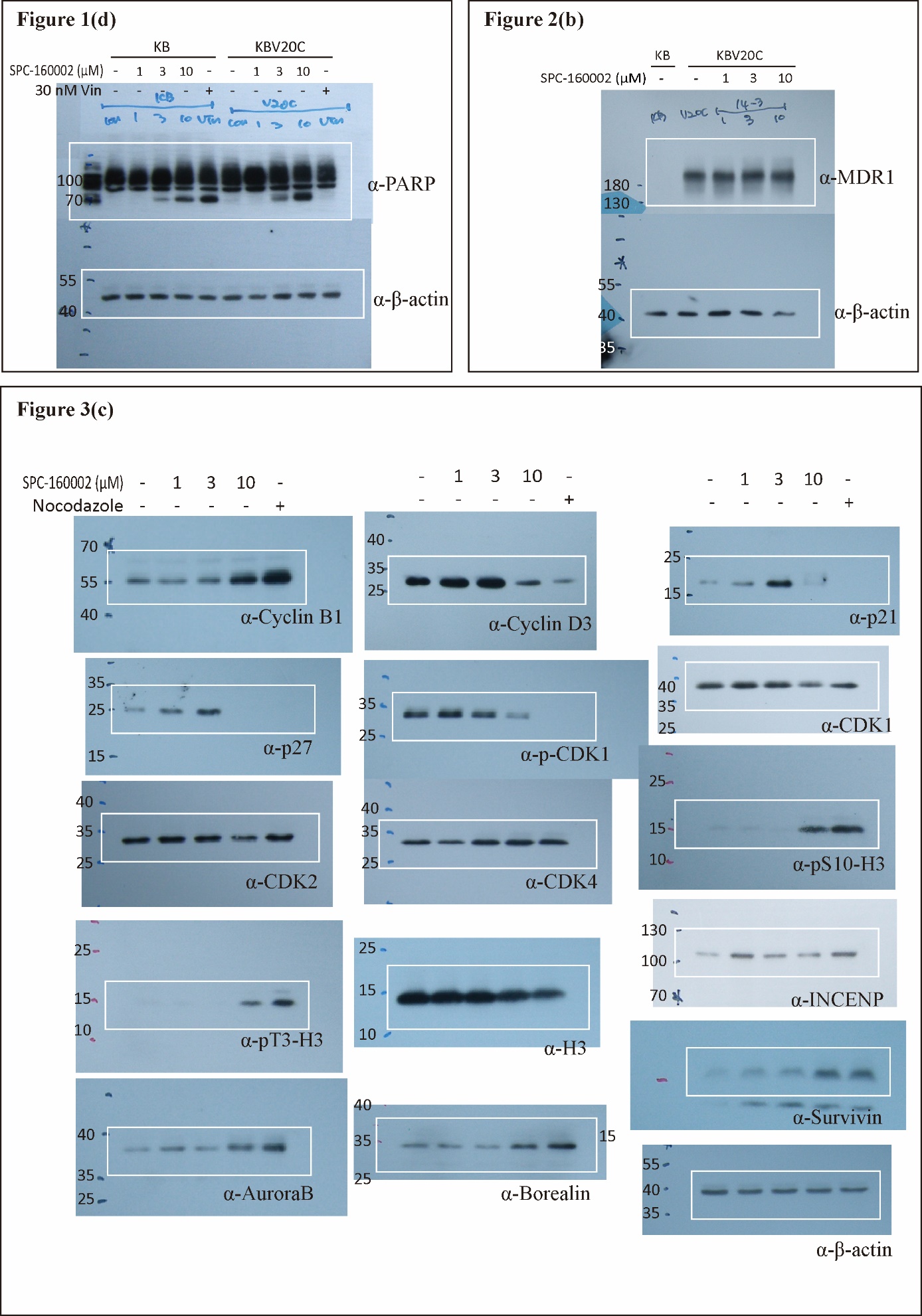


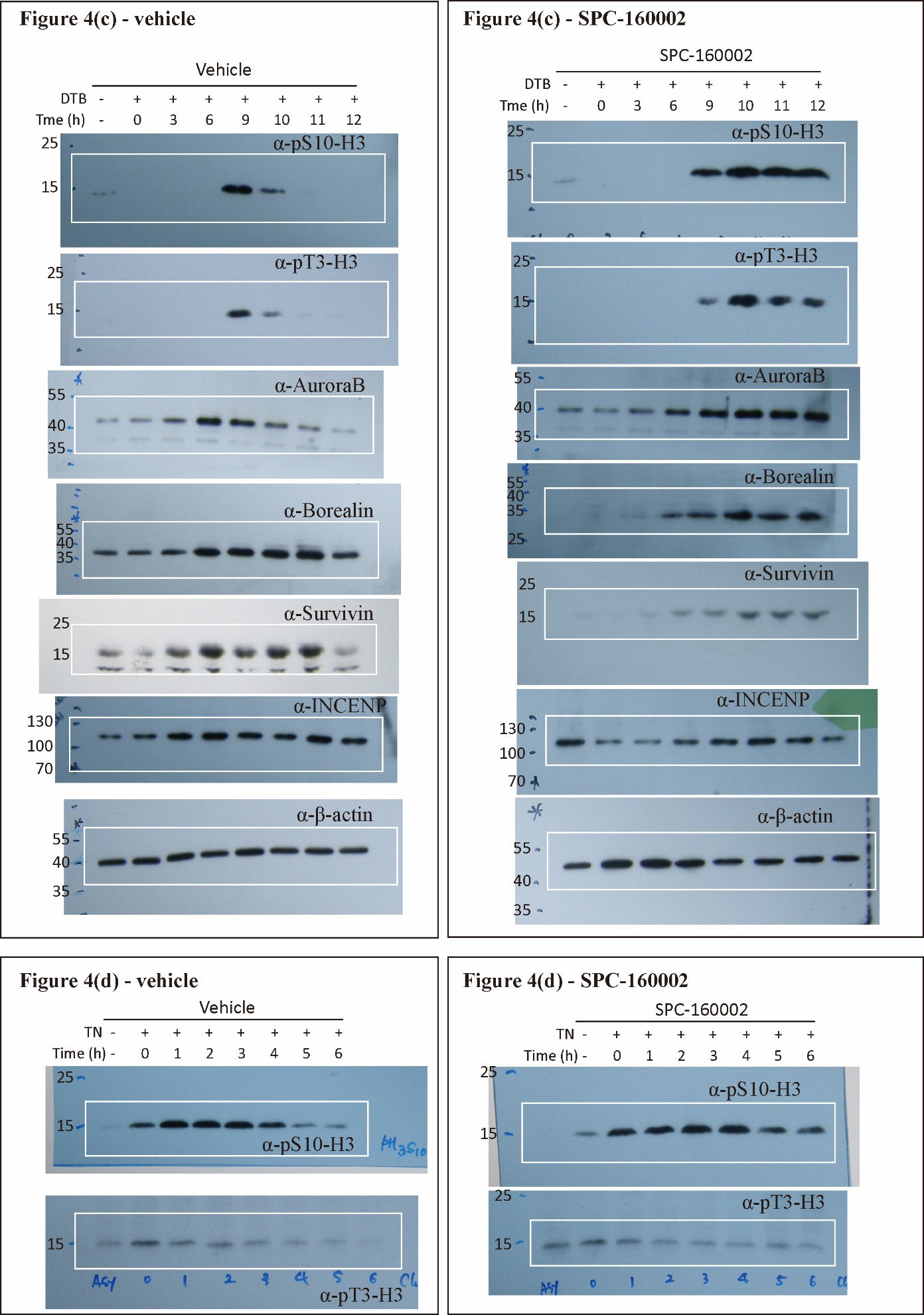


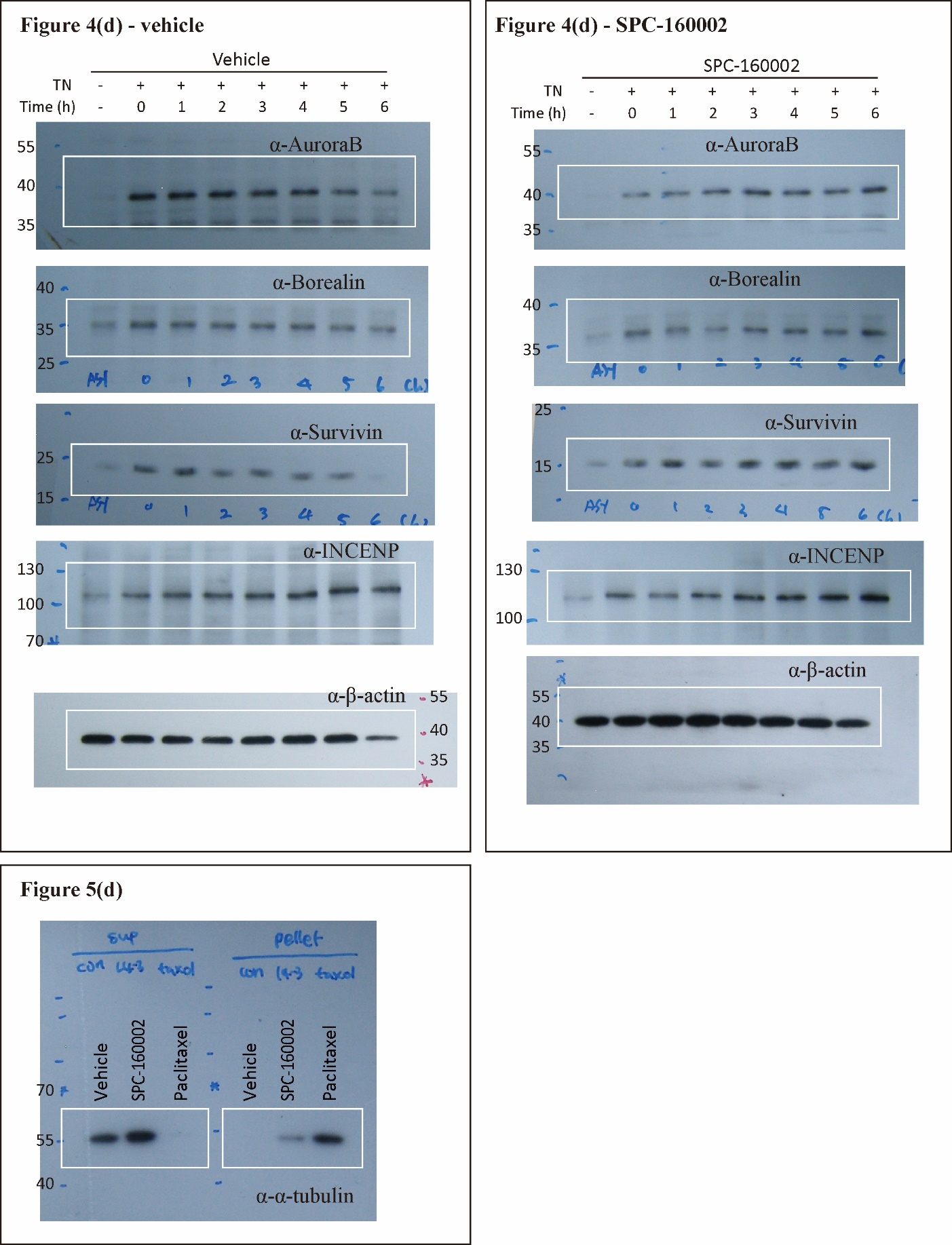

Supplement: Supplementary file 1 — Supplementary Information. [file 41598_2021_90337_MOESM1_ESM.docx]
